# Characterizing free-living and particle-attached bacterial communities of a canyon river reservoir on the Yungui Plateau, China

**DOI:** 10.3389/fmicb.2022.986637

**Published:** 2022-08-31

**Authors:** Yang Yang, Chen Chen, Junyi Wang, Tao Xu

**Affiliations:** ^1^School of Life Sciences, Guizhou Normal University, Guizhou, China; ^2^Guizhou School of Emergency Management, Guizhou Normal University, Guizhou, China

**Keywords:** river reservoir, aquatic bacteria diversity, community composition, network features, environmental drivers

## Abstract

Revealing the composition of free-living (FL) and particle-attached (PA) bacterial communities could provide insights into their distinct roles in biogeochemical processes and algal bloom dynamics. While there is still a lack of research about the difference and interactions between FL and PA communities, especially on the Yungui plateau with underestimated diversity. This study unveiled the structure of both FL and PA bacterial communities in a canyon reservoir (Wujiangdu) on the Yungui Plateau, southern China. Water samples were collected from surface water at nine sites in the reservoir. FL and PA bacterial community structures were identified by high-throughput 16S rRNA gene sequencing. We compared the structure and diversity of FL and PA bacteria and investigated their relationship with environmental factors. Results showed that there were different structures between FL and PA bacterial communities, and the dominant FL and PA phyla were affected by different environmental variables. Moreover, diversity of PA bacteria was greater than that of FL bacteria. Both groups exhibited distance decay patterns in this reservoir with varying correlations with geographic distances. FL fraction, however, exhibited a stronger correlation with environmental factors than the PA counterpart. Both FL and PA communities were phylogenetic clustering than expected according to the mean nearest taxon distance. This study provides fundamental information on FL and PA bacteria distribution and demonstrates how specific environmental factors affected these two bacterial fractions in canyon river reservoirs.

## Introduction

Bacteria act as sentinels of environmental changes due to their sensitivity to trophic status and anthropogenic pollution (Harnisz, 2013; Savio et al., 2015). Based on their lifestyle, there are two bacterial fractions, i.e., free-living (FL), with a size range between 0.22 and 5 μm (Grossart, 2010; Crespo et al., 2013), and particle-attached (PA) larger than 5 μm. FL refers to bacteria floating freely in the water column, while the PA fraction comprises bacteria attached to organic particles and living organisms. FL and PA bacteria have different ecological roles with distinct dispersal potentials and adaptive strategies. Several studies revealed that there were significant phylogenetic differences between FL and PA fractions, whereby some bacteria can rapidly exchange between the size fractions (Bizic-Ionescu et al., 2015; Tang et al., 2015). However, whether PA is distinct from FL remains uncertain owing to the complex lifestyle of aquatic bacteria. It has been demonstrated that PA bacteria could also survive freely in the water column and subsequently colonize new particles. This implies that PA bacteria, sometimes, contribute to the FL bacterial community (Crespo et al., 2013). Studies suggested that these two groups differed in structure in the open sea, but are similar in rivers and turbid estuaries (Ortega-Retuerta et al., 2013). Tang et al. (2015, 2017) has suggested high connectivity between the FL and PA fractions. Therefore, unveiling the difference and interactions between FL and PA bacteria could help us understand their functions in biogeochemical processes in aquatic ecosystems.

Bacterial research on river reservoirs on Yungui-Plateau is scarce, particularly the roles of FL and PA fractions. Plateau lakes are relatively isolated and sensitive to anthropogenic activities, and the microbial diversity in these habitats is underestimated. Only a few studies reported the bacterial community structure in the Yungui-Plateau region, and they rarely separated the FL and PA fractions. Zhang et al. (2020) investigated the relationship between phytoplankton and bacterioplankton in Dianchi Lake, which was mainly dominated by *Proteobacteria* and *Bacteroidetes.* Wu et al. (2019) studied the vertical profile of the sediment bacterial community in Lake Lugu and suggested that chemical variables were the main drivers of dominant phyla. Recently, Yue et al. (2021) investigated the vertical distribution of the bacterial community in the Wujiangdu reservoir and emphasized the role of stratification on the vertical pattern of bacterial community structure. It was observed that *Proteobacteria*, *Actinobacteria,* and *Bacteroidetes* were the predominant phyla in the water column, with decreasing abundance from surface to bottom. Wujiang River is the largest southern tributary of the Yangtze River and also the largest river in Guizhou Province. Wujiangdu reservoir was the first major dam established in the middle and upper reaches of the Wujiang River. Due to the karstic characteristics of the catchment area, the water is rich in carbonates from weathering. Yet, the influence of such environmental conditions o microbial composition, diversity, and activities remain unknown. Frequent cascade dams along the Wujiang River potentially affect the overall microbial community *via* alteration of hydrological features. A recent study found that bacteria prefer an FL lifestyle in nutrient-rich water (Dang and Lovell, 2016). Thus, research on aquatic microbial communities should be placed in the context of the relationship between FL and PA bacterial communities. The differences between these two groups are critical to reveal the elemental recycling and metabolic pathways in aquatic ecosystems. Until now, the community structure and function of FL vs. PA bacteria, in such plateau reservoirs remain poorly understood. Information about bacterial distribution, assembly, diversity, and functioning in this reservoir could help to better understand the mechanisms underlying microbial food web and phytoplankton dynamics in these man-made freshwater ecosystems.

We sequenced the 16S rRNA marker gene of surface water samples collected from 9 locations to simultaneously investigate the FL and PA bacterial community structure in this understudied canyon river reservoir. In particular, we aimed to describe the structure of both FL and PA bacterial community structures and unveil the environmental factors controlling these two fractions. We hypothesized that (1) FL and PA fractions differ in structure and diversity; and (2) FL and PA bacteria were affected by different environmental factors, implying their distinct assembly mechanisms in this riverine ecosystem. This study could pave the way for further research on microbial communities in the Wujiangdu reservoir.

## Materials and methods

### Location information, field sampling, and water chemical analysis

Wujiangdu Reservoir, built in 1979, is located in the lower basin of the Wujiang river, and at the conjunction of Xifeng town and Zunyi city, Guizhou Province. It has a watershed of 2.78 × 10^3^ km^2^, a mean depth of 154 M, and a volume of 23 × 10^8^ M^3^. The average water residence time is 53 days. This reservoir has a history of cage aquaculture activities since 1999.

**Figure 1:**
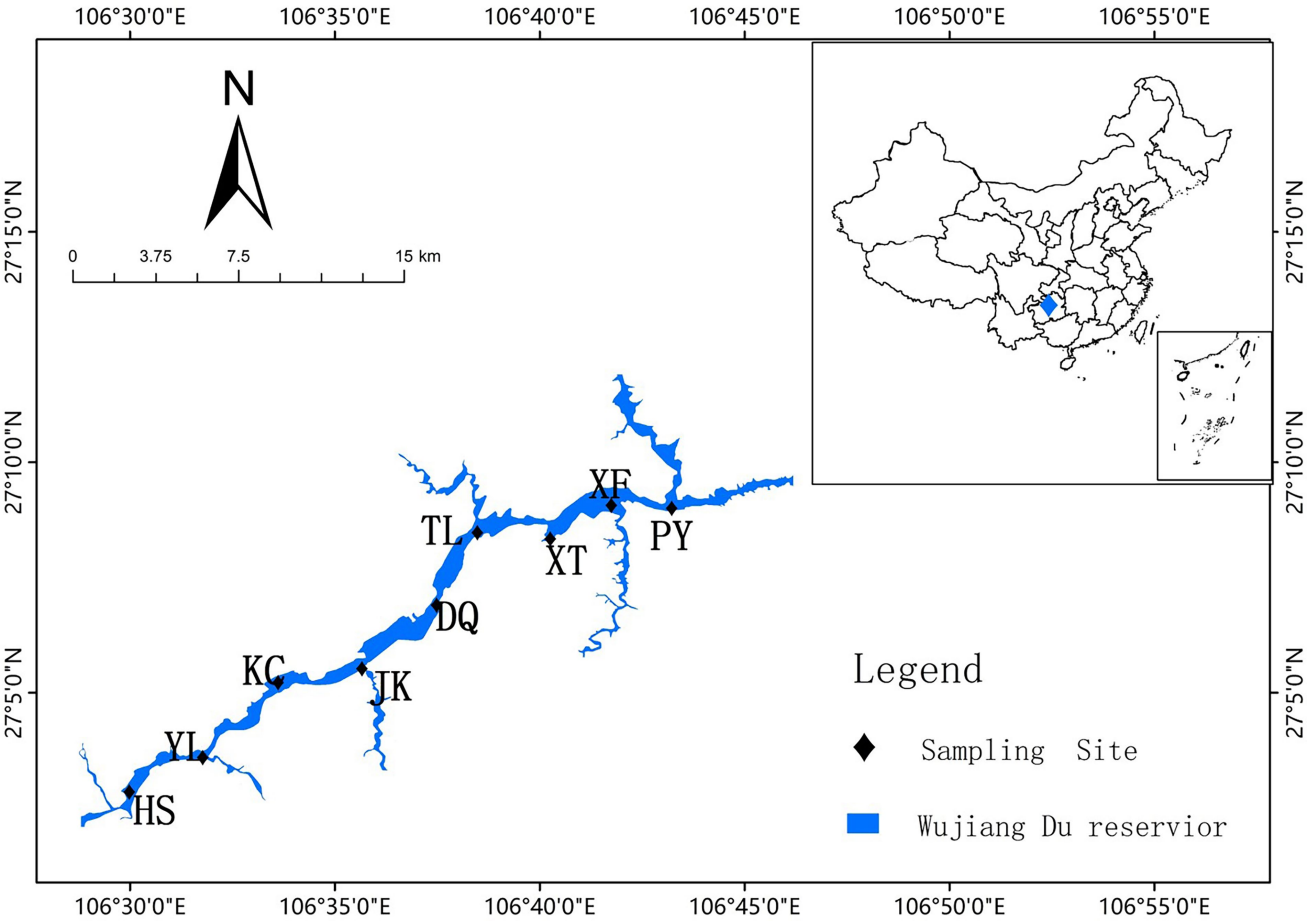
Sampling locations of Wujiangdu Reservoir along the Wujiang River.

On August 19th, 2021, field sampling was conducted at 9 locations (HS, YL, KC, JK, DQ, TL, XT, XF, and PY) along the river in Wujiangdu Reservoir (Figure 1, map created using ArcGis 10.8). These locations were chosen due to their position at the confluence of small rivers and the main river. At each location, water temperature, Dissolved Oxygen (DO), pH, and Conductivity (Cond) were measured on-site *via* a portable YSI probe (HANNA HI98194). Secchi depth (SD) was measured by a Secchi disk at each station. Surface water (0.5 m) samples were taken using a 5 l Schindler sampler and then transported back to the lab in a dark container. 1 L of water was used for water chemical analysis, and an additional 1 L was filled in a sterile PE bottle which was kept at site temperature for transport to the lab. For bacterial DNA, 400 ml water was filtrated with 5.0 μm polycarbonate filters (PA fraction), and then filtered through 0.22 μm filters (FL fraction). All filters were stored at −80°C for further analyses.

Total phosphorus (TP), phosphate (PO_4_^3−^), total nitrogen (TN), nitrate (NO_3_^−^), nitrite (NO_2_^−^), silicate (SiO_2_), and Chlorophyll a (Chl *a*) were measured according to standard methods (A.P.H.A, 2012). Based on these measurements, the trophic status index (TSI) was calculated according to the equations below (Carlson, 1991; An and Park, 2003). It indicates a different trophic status by values as oligotrophic (<40), mesotrophic (40 to 50), eutrophic (50 to 70), and hypereutrophic (>70).

TSI_SD_ = 60–14.42 ln (SD).

TSI_TN_ = 54.45 + 14.43 ln (TN).

TSI_TP_ = 14.42 ln (TP) + 4.15.

TSI_CHL_ = 9.81ln (CHL) + 30.6.

### Bacterial sequencing and statistical analyses

Genomic DNA was extracted using the MagaBio Soil and Feces Genomic DNA Purification Kit (Bori Technology, China) according to the manufacturer’s instructions. V4-V5 region of the 16S rRNA gene was amplified using the primer 515F (5’-GTGCCAGCMGCCGCGGTAA-3′) and reverse primer 907R (5’-CCGTCAATTCMTTRAGTTT-3′). Amplification was undertaken *via* PCR in triplicates by BioRad S1000 (Bio-Rad laboratory, CA). No negative controls were assessed from DNA extraction to sequencing. PCR reactions, containing 25 μl 2x Premix Taq, 1 μl each primer, and 50 ng DNA in a volume of 50 μl were amplified by thermocycling: 5 min at 94°C for initialization; 30 cycles of 30 s denaturation at 94°C, 30 s annealing at 52°C, and 30 s extension at 72°C, followed by 10 min final elongation at 72°C. Then triplicates/sample of PCR products were combined with the length and concentration detected by 1% agarose gel electrophoresis. PCR products were mixed in equi-density ratios according to the GeneTools Analysis Software (Version4.03.05.0, SynGene). Based on the NEBNext Ultra™ DNA library Prep kit for Illumina standard protocol, a library was established before sequencing on an Illumina Nova 6,000 platform and 250 bp paired-end reads were generated. Pair-end raw reads filtering was performed according to fastp (an ultra-fast all-in-one FASTQ preprocessor, version 0.14.1). Sequences were assigned to each sample based on their unique barcode and primer, after which the barcodes and primers were removed and got the pair-end clean reads. Then raw tags were constructed using usearch-fastq_mergepairs (V10) based on the relationship of the overlap between the pair-end reads with the minimum overlap length of 16 bp, and the maximum allowable error overlap region of 5 bp. Quality filtering on the spliced sequences was performed using fastp to obtain effective clean tags. Then16S rRNA gene sequences were analyzed by Usearch software (V8.0.1517), and sequences with ≥97% similarity were assigned to the same OTU. Representative sequences for each OTU were screened for further annotation. The singleton OTU, generally regarded as obtained from sequencing errors, or chimeras was removed using usearch after the OTU cluster. The chimer sequences were detected and removed using the UCHIME de nova algorithm. Raw reads are stored in the NCBI short read archive with the accession: PRJNA858177. For each representative sequence, the GreenGene database was used based on the RDP classifier algorithm and the assign_taxonomy.py script in Qiime to annotate taxonomic information. OTU and its tags, which are annotated as chloroplasts or mitochondria were removed. Then OTU taxonomy synthesis information table and number of effective tags were obtained. OTU abundance data was rarefied using a standard sequence number corresponding to the sample with the least sequences. Subsequent analyses of alpha diversity and beta diversity were all performed based on this rarefied data. The rank abundance curve of FL and PA was plotted in Supplementary Figure S1.

The OTUs present in at least 7 samples of FL and PA groups, separately, were selected to construct the network using the molecular ecological network analysis (MENA) pipeline. Relative abundance of OTUs was used for Pearson correlation coefficients. The Random Matrix Theory was applied to determine the transition point of the nearest-neighbor spacing distribution of eigenvalues from Gaussian to Poisson distribution. The transition point was regarded as the threshold for network construction. FL and PA group networks were constructed based on the threshold of 0.95. The topological roles of each OTUs can be identified by Zi and Pi values. Zi refers to the connectivity of node i within modules, whereas Pi is the connectivity of node i among nodules (Guimera and Amaral, 2005). According to Pi and Zi values, the nodes could be classified into four categories: (1) peripherals (Zi ≤ 2.5, Pi ≤0.62); (2) connectors (Zi ≤ 2.5, Pi >0.62); (3) Module hubs (Zi > 2.5, Pi ≤0.62); (4) network hubs (Zi > 2.5, Pi >0.62; Zhou et al., 2011).

Diversity indices were calculated in R, including OTU Chao1, Abundance-based coverage estimator (ACE), and Simpson index. Chao1 and ACE are used to estimate richness. Simpson indexes are mostly influenced by evenness, taking into account both richness and abundance. The bigger the value, the lower the diversity. In addition, β-diversity was calculated using Bray–Curtis dissimilarity. Phylogenetic diversity (PD) was measured to estimate phylogenetic relatedness in the community. Analysis of variance (ANOVA) tests were used to test for significant differences in the relative abundance of dominant taxa and diversity between FL and PA fractions. Permutational multivariate analysis of variance (PerMANOVA) was used to test whether there was a significant difference in community composition between groups (FL vs. PA; Anderson, 2008). The distance decay pattern was tested using Mantel tests, which calculate geographic distance based on sampling coordinates, and environmental parameters using Euclidean distance. Redundancy analysis (RDA) was used to summarize the relationship between bacterial community and measured environmental factors. PerMANOVA, PERMDISP, and RDA were performed using the ‘vegan’ package in R (Oksanen et al., 2020). The mean nearest taxon distance (MNTD) evaluated the mean distance separating each taxon in the community from its closest relative. The standardized effect size of MNTD (ses.MNTS) describes the difference between phylogenetic distances in the observed communities versus null communities generated with randomization. MNTD and ses.MNTD were calculated to assess whether FL and PA communities were phylogenetic evenness or clustering using the ‘picante’ package (Kembel et al., 2010).

## Results

### Water physio-chemical parameters

Water transparency, indicated by SD, ranged from 2.0 to 3.9 M. TN concentration ranged from 2.38 mg/l to 3.25 mg/l, and TP from 0.015 mg/l to 0.019 mg/l. NO_3_^−^ was the major component of dissolved nitrogen, ranging from 1.78 mg/l to 2.54 mg/l, followed by NO_2_^−^ (0.009 mg/l-0.030 mg/l) and NH_4_^+^ (0.009 mg/l-0.030 mg/l). The concentrations of TN, TP, PO_4_^3−^, NO_3_^−^, Chl *a,* and TSS could be seen in Supplementary Figure S2. Trophic Status Index (TSI) was calculated according to the equation mentioned above (Table 1).

**Table 1:** Summary of Trophic Status Index (TSI) in Wujiangdu reservoir.

TSI	Mean	Min	Max
TSI_SD_	46.66	40.37	50.00
TSI_TN_	68.81	66.97	71.47
TSI_TP_	44.79	43.31	46.76
TSI_CHL_	49.55	43.09	56.62

### FL and PA bacterial communities in surface water

**Figure 2:**
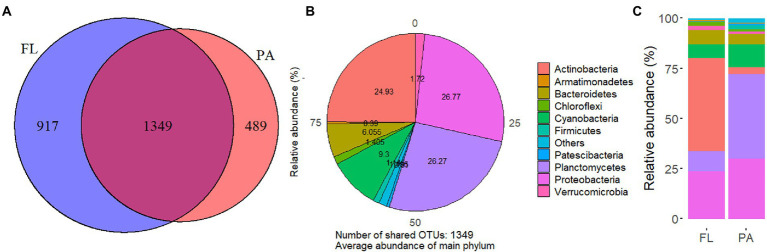
Venn diagram at OTU level in FL and PA **(A)**, and the distribution of shared OTUs at phylum level in both groups **(B)**, and relative abundance of main phyla in FL and PA **(C)** in Wujiangdu reservoir.

In total, 772,264 and 764,333 clean reads were obtained after trimming and quality control for FL and PA samples, respectively. The number of sequences produced from FL and PA samples was 591,496 and 465,514, respectively. 1,349 OTUs were shared by FL and PA fractions, with 489 unique OTUs in FL and 917 unique OTUs in PA (Figure 2A). The detected bacterial OTUs belonged to 37 phyla and 72 classes. These shared OTUs are distributed into 10 major phyla with varying relative abundance in FL and PA (Figure 2B). Dominant phyla in FL include *Actinobacteria* and *Proteobacteria*, while dominant phyla in PA were *Planctomycetes* and *Proteobacteria* (Figure 2C).

At the class level, *Actinobacteria* and *Acidimicrobiia* accounted for 46.5% of total abundance in FL. *Planctomycetes* predominated (42.4%) in PA, followed by α-*Proteobacteria* (17.8%) and *ϒ-Proteobacteria* (11.6%). ANOVA tests showed significant differences in the relative abundance of *Actinobacteria* and *Planctomycetes* between FL and PA. On order level, *Frankiales* (31.7%) and *Microtrichales* (13.5%; *Actinobacteria*) were dominant in FL, whereas *Pirellulales* (*Planctomycetes*) predominated (38.8%) in PA. At the family level, the dominant groups in the FL fraction were *Sporichthyaceae*, *Illumatobacteraceae* (*Actinobacteria*), and *Burkholderiaceae* (Proteobacteria). Whereas *Pirellulaceae* (*Planctomycetes*) dominated the PA bacterial communities.

From the perspective of community composition, perMANOVA suggested that there were significant differences between FL and PA bacterial communities at OTU, genus, and phylum levels (*p* = 0.001). Simultaneously, PERMDISP tests showed insignificant homogeneity of variances of FL and PA (*p* = 0.22). SIMPER analysis showed that significantly different phyla between FL and PA fractions were *Actinobacteria* and *Planctomycetes* (*p* = 0.001). Indicator species analysis on phylum level suggested that *Spirochates* was the indicator taxa in FL whereas *Thaumarchaeota* was indicative in PA (*p* < 0.01).

**Figure 3:**
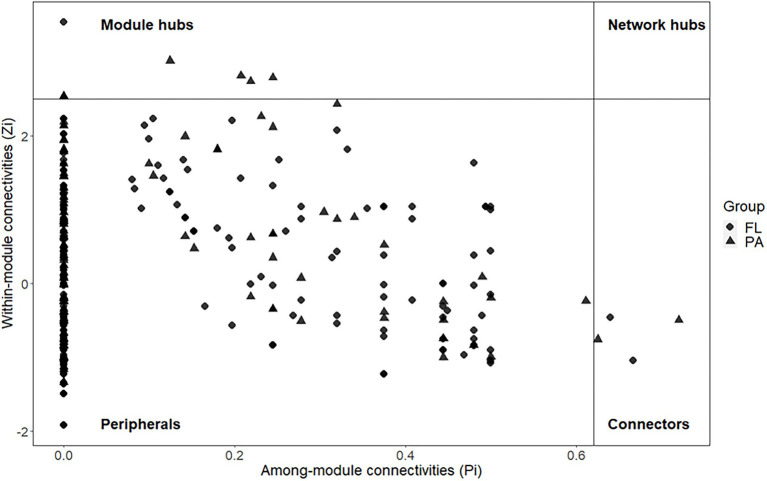
Z-P plot showing the distribution of OTUs based on their topological features in FL and PA bacterial communities.

There were 244 nodes with 685 links in the FL network, while 223 nodes and 473 links were observed in the PA network. PA network had stronger modularity than the FL network (0.749 > 0.675). The topological roles of the OTUs identified in FL and PA were shown in Figure 3, with the details about module hubs and connectors shown in Table 2. Most of the OTUs (99.0% for FL and 97.1%) for PA were peripherals, which possess most of their links inside their modules. Among the peripheral OTUs, 71.7 and 86.8% in FL and PA networks, respectively, had no links at all with other modules (Pi = 0). One module hub and two connectors were detected in the FL network. While two module hubs with six connectors were present in the PA network. However, no network hubs were observed in either network. The topological features of the FL and PA network indicated different roles of individual roles in the network interactions.

**Table 2:** Module hubs and connectors in the Network analysis of FL and PA groups.

	No. of module	Node degree	OTU	Phylum
**Module hubs**	FL (F1)	11	OTU_1082	*Bacteroidetes*
	PA (P3)	15	OTU_928	*Planctomycetes*
	PA (P2)	17	OTU_159	*Bacteroidetes*
	PA (P2)	14	OTU_2318	*Proteobacteria*
	PA (P1)	8	OTU_2673	*Proteobacteria*
	PA (P7)	5	OTU_289	*Bacteroidetes*
	PA (P5)	7	OTU_741	*Proteobacteria*
**Connectors**	FL (F1)	5	OTU_240	*Armatimonadetes*
	FL (F5)	3	OTU_2788	*Actinobacteria*
	PA (P4)	4	OTU_581	*FBP*
	PA (P3)	5	OTU_362	*Bacteroidetes*

### Bacterial community diversity

**Figure 4:**
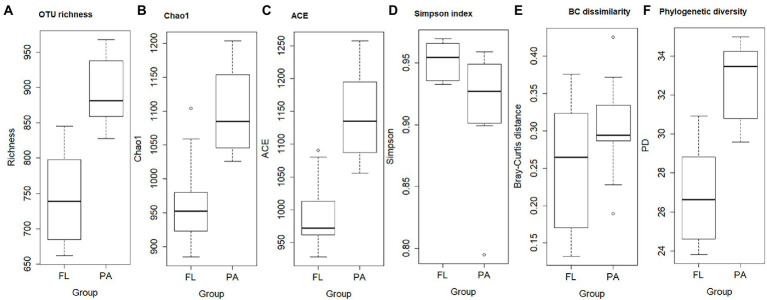
Boxplot of α-diversity indexes (**A**. OTU richness; **B**. Chao1; **C**. ACE; D. Simpson), β-diversity (Bray–Curtis dissimilarity) **(E)**, and phylogenetic diversity (PD) **(F)** of FL and PA bacterial community.

**Figure 5:**
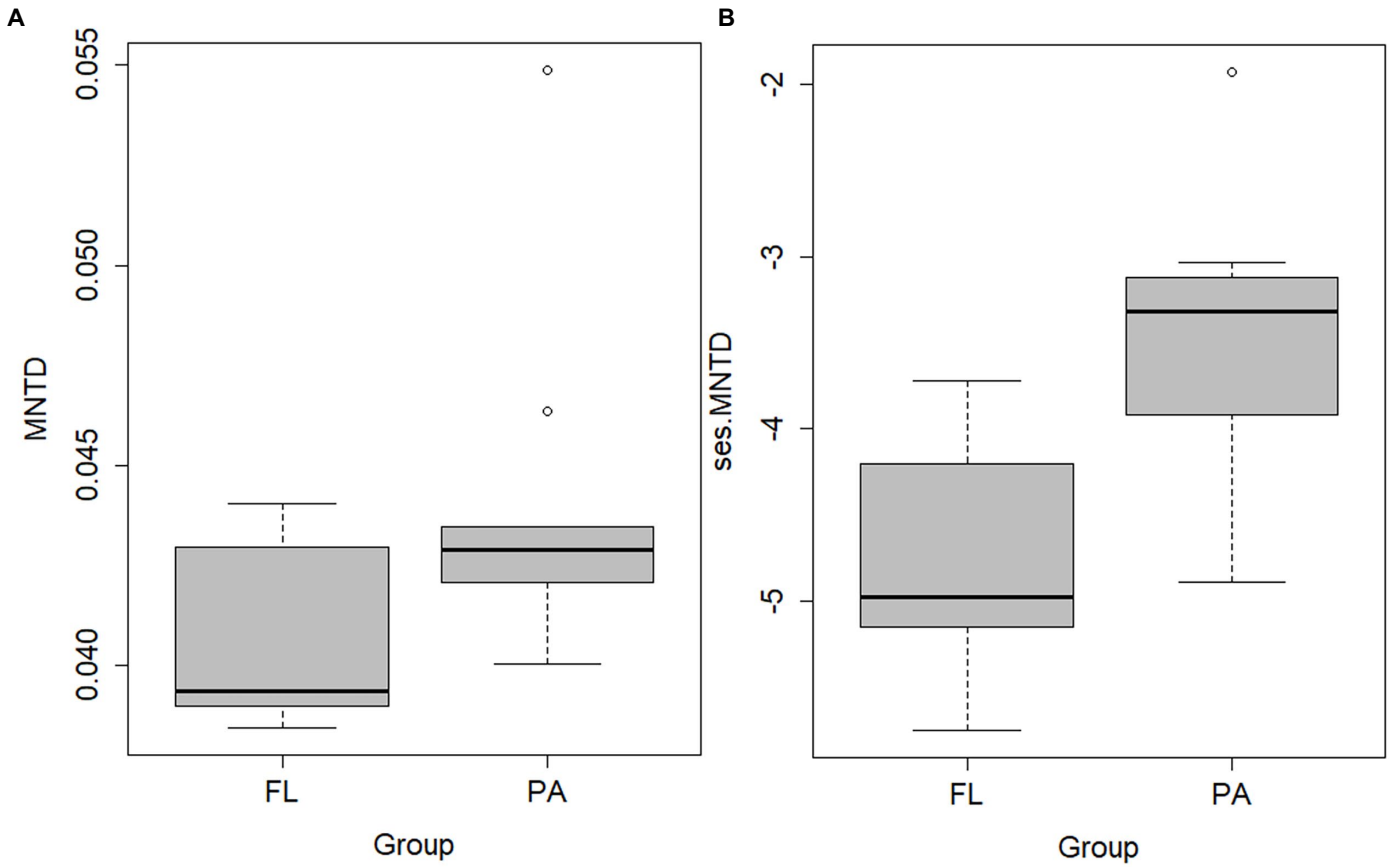
MNTD **(A)** and the standardized -effect size of MNTD (ses.MNTD); **(B)** of the FL and PA bacterial communities.

The OTU richness, Chao1, and ACE were greater in PA than that in FL (Figures 4A–C). The greater the Chao1 or ACE indices, the higher the expected species richness of the community. Simpson indices, however, were lower in PA than that in FL (Figure 4D). BC dissimilarity and PD were greater in PA than in FL, indicating higher diversity in PA than in FL bacteria (Figures 4E,F). Significant differences were detected between FL and PA bacterial communities on OTU richness, Chao1, ACE indexes, BC dissimilarity, and PD (*p* < 0.01). In addition, MNTD and ses.MNDT were computed for FL and PA bacterial communities. Both values were lower in FL than in PA (Figure 5).

### Relationship between environment and bacterial community structure

**Figure 6:**
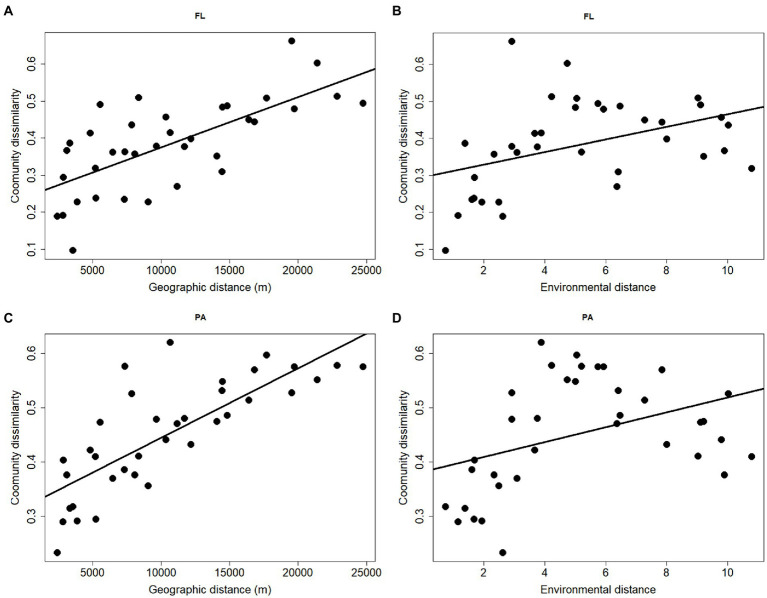
Relationship between geographic distance, environmental distance and Bray–curtis dissimilarity of FL **(A,B)** and PA **(C,D)** bacterial communities in Wujiangdu Reservoir.

Mantel tests showed that there were significant effects of geographic distance and environmental factors on FL bacterial community (env: *r* = 0.46, *p* = 0.01; geo: *r* = 0.69, *p* = 0.001). On the other hand, only weak significant effects of environmental factors were detected on PA bacterial community (env: *r* = 0.45, *p* = 0.02), but significant effects of geographic distance were observed on PA fraction (geo: *r* = 0.82, *p* = 0.001; Figure 6). In addition, the results showed that geographic distance exhibited stronger correlations with PA bacterial communities than FL, as indicated by the higher r values.

**Figure 7:**
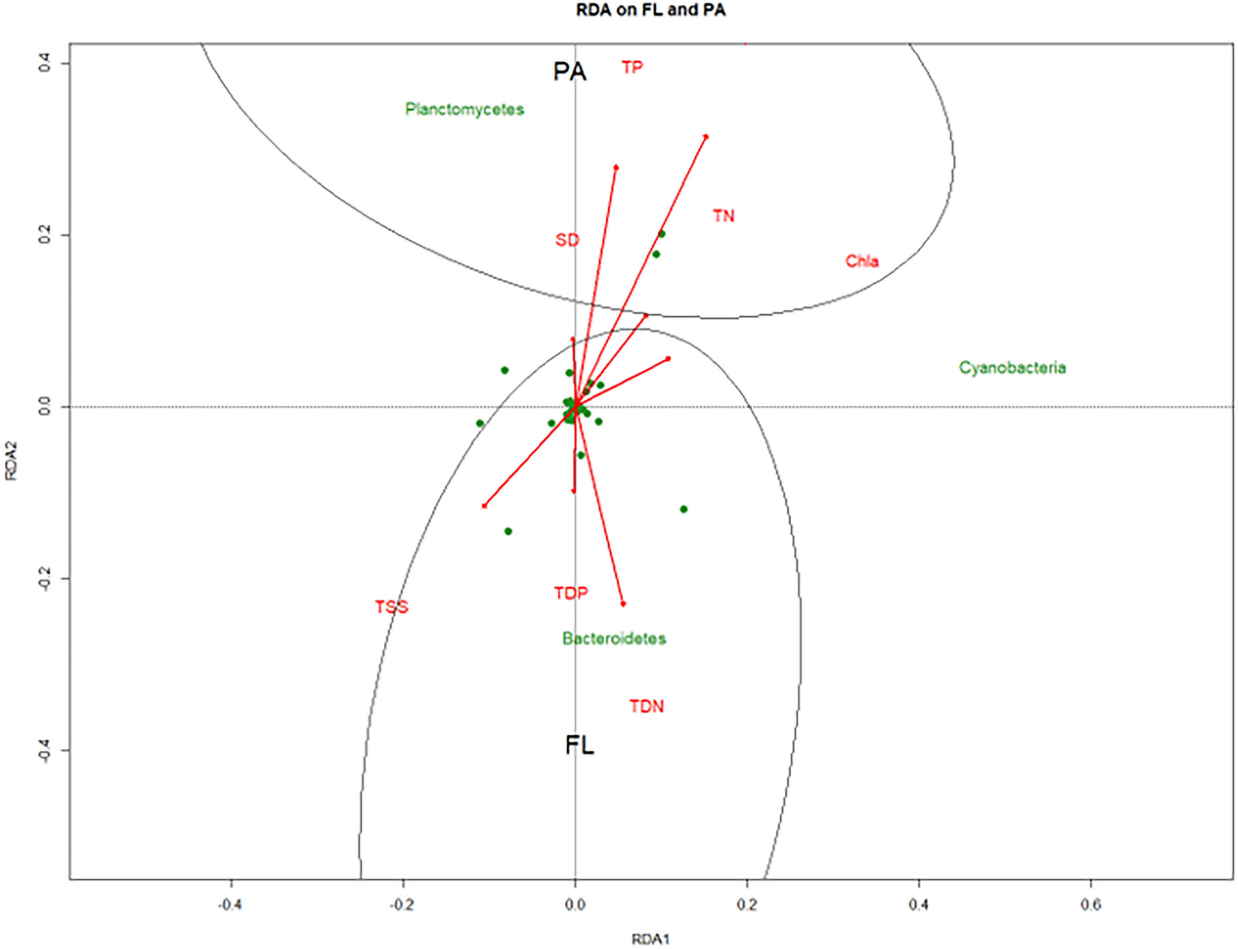
RDA plots of FL and PA bacterial community in Wujiangdu reservoir.

RDA was performed to reveal the relationship between environmental variables and FL vs. PA bacterial communities at the phylum level. FL and PA exhibited dissimilar structures as indicated by a certain extent of the distance between these two groups on the plot. FL fraction showed positive correlations with TDN, TDP, and TSS, whereas PA fraction was associated with SD, TN, TP, pH, and Chl *a* (Figure 7).

## Discussion

Based on TSI_TN_ values, the Wujiangdu reservoir was eutrophic, with a maximum value above the eutrophic threshold. Whereas it was mesotrophic based on TSI_SD_, TSI_TP,_ and TSI_CHL_ with maximum values close to the threshold of eutrophic. There has been a long history of fish cage culture in this reservoir (since 1999) and the application of plant fertilizer in the catchment area, both of which contributed to the increasing trend of eutrophication. Based on water chemical variables, there was high spatial heterogeneity in the reservoir. This might be attributed to the multiple influents of tributaries, which were affected by varying anthropogenic pollution, such as agricultural activities, fertilizer plants, and fishing.

This study was the first report concerning the FL and PA bacterial structure in the Wujiangdu reservoir. The predominant bacterial groups in this reservoir were *Proteobacteria* and *Actinobacteria*. This observation is consistent with one study from this reservoir, which did not separate FL and PA (Yue et al., 2021). Other studies on plateau lakes also reported the predominant role of *Proteobacteria* and *Actinobacteria* in the water column (Wu et al., 2019; Zhang et al., 2020). Both groups constitute frequent and highly abundant bacterial members in eutrophic lakes (Trusova and Gladyshev, 2002). *Burkholderiaceae* (*Proteobacteria*) are aerobic chemoorganotrophs (Coenye and Vandamme, 2003). *Sporichthyaceae*, members of *Frankiales*, are less dependent on high carbon and nutrient concentrations due to their facultative aerobic lifestyle (Garcia et al., 2013). In addition to their light-driven metabolism, this group also possesses nitrogen fixation capabilities and can utilize cyanobacterial biomass as an energy source. Such physiological features provide them great advantages in variable aquatic environments (Garcia et al., 2013; Normand and Fernandez, 2020). The most abundant genus of *Sporichthyaceae* was the hgcI_clade, also known as acI cluster, which constitutes the main bacterial group in many freshwater ecosystems (Warnecke et al., 2005).

The significant indicator phylum in the FL fraction was *Actinobacteria*, whereas in the PA fraction was *Planctomycetes*. *Actinobacteria* are highly resistant to protistan grazing due to their reduced cell size and cell wall type, leading to their prevalence in freshwater ecosystems (Newton et al., 2011). Moreover, *Actinobacteria*, known to be able to accumulate phosphorus (P) as polyphosphate within the cell (Seviour et al., 2003), has advantages in a P-depleted environment. Compared with *Actinobacteria*, *Planctomycetes*, known as particle-degraders, were abundant in PA fraction. This was due primarily to their preference for particulate organic matter. The potential role of *Planctomycetes* in nitrogen and carbon biogeochemical cycles has been reported, including their presence in activated sludge with anaerobic metabolism (Chouari et al., 2003). Studies noticed their changes during or after phytoplankton or cyanobacterial blooms (Pizzetti et al., 2011). Their co-occurrence with cyanobacteria in our observation might be the clue for the ensuing cyanobacterial blooms. FL and PA bacteria were associated with various environmental factors, with FL mainly relevant to dissolved nutrients, such as TDN, and TDP, whereas PA bacteria were primarily related to particles in the water column.

Both module hubs and connectors play important and different roles in the ecological network. According to their definitions, the extinction of a module hub may result in disassembling of its module, with less or no effects on other modules. The disappearance of a connector, however, may lead to the disassembling of the entire network into isolated modules, but without effects on the inner structure (Olesen et al., 2007). Our results indicated the distinct roles of individual nodes within the community interactions. *Bacteroidetes* were observed in both module hubs and connectors. They played an important role as a module hub in the FL network, whereas they were present as connectors in the PA network. Module hubs in PA bacterial community ensured that species within a module were linked tightly, as showed by the stronger modularity in the PA group than in the FL counterparts. More connectors in the PA network also provided evidence of different network structures between FL and PA fractions.

Both α-diversity and β-diversity were higher in the PA than the FL fraction, suggesting that PA communities were more diverse than FL bacteria. Earlier, it has been proposed that the similarity between FL and PA bacterial communities depended on the contents of organic matter in aquatic ecosystems (Hu et al., 2020). Studies suggested that trophic status was associated with the complexity and composition of FL and PA bacteria in mesotrophic vs. eutrophic lakes (Parveen et al., 2011; Tang et al., 2015). The generally higher diversity of PA than FL bacteria results from differences in available organic carbon on particles vs. the water column. PA bacteria, attached to phytoplankton or suspended particles in the water column, are parts of the phyco- or detritosphere. The phycosphere constitutes highly specific micro-habitats, selecting for species with specific metabolic capabilities such as high enzymatic activities. The generally higher heterogeneity of particles in comparison to dissolved organic matter determines the observed greater diversity of the PA bacterial community.

The bacterial community structure in Wujiangdu Reservoir exhibited pronounced spatial heterogeneities along sampling locations, as evidenced by Mantel tests. Compared to the FL fraction, the PA community composition exhibited a greater extent of heterogeneity. According to the metacommunity theory, bacteria with strong dispersal capacity should be able to thrive at every location. Stochastic processes, however, could also involve the community assembly. Thus, the observed spatial heterogeneity can be seen as the results of various species sorting mechanisms plus stochastic processes. The negative values of ses.MNTD indicated phylogenetically clustered communities than expected for both FL and PA bacteria, which suggested that both fractions were affected by environmental filtering. The smaller ses.MNTD in FL fraction implies that environmental filtering was stronger than PA. The presence and absence of one taxon depend on the specific environmental conditions acting as a filter for the best-adapted taxon. This implies that the observed differences in community structure between sampling stations were mainly affected by the local environmental conditions, including physical, chemical, and biological conditions, such as hydrodynamics and nutrient status, but also food web characteristics. This is consistent with the famous tenet ‘everything is everywhere but the environment selects’ (Bass-Becking, 1934).

## Conclusion

The reservoir shows the potential of increasingly eutrophied due to the manifold anthropogenic activities, such as industry, tourism, and farming. Based on bacterial lifestyle, FL and PA fractions exhibited distinct differences in community structure, diversity, and gene functions. FL bacteria were mainly affected by the dissolved nutrients. Whereas PA was associated with the particles in the water column. This study adds to our knowledge of the diversity and functionality of FL vs. PA bacterial communities in reservoirs under high anthropogenic pressure.

## Data availability statement

The datasets presented in this study can be found in online repositories. The names of the repository/repositories and accession number(s) can be found at: NCBI, PRJNA858177.

## Author contributions

YY contributed to the study conception and design. Material preparation, data collection, and analysis were performed by CC, JW, and TX. The first draft of the manuscript was written by YY and TX. All authors contributed to the article and approved the submitted version.

## Funding

This study was funded by the National Natural Science Foundation of China (No.32060270), the Planning Project for Science and Technology in Guizhou [(2020)1Y072], the Project for Innovation and entrepreneurship of high-level overseas talents in Guizhou [Grant No. (2020)09], and the Ph.D. start-up project of Guizhou Normal University (11904-0519087). 

## Acknowledgments

We appreciate Professor Hans-Peter Grossart (Leibniz-Institute of Freshwater Ecology and Inland Fisheries) for comments on this manuscript.

## Conflict of interest

The authors declare that the research was conducted in the absence of any commercial or financial relationships that could be construed as a potential conflict of interest.

## Publisher’s note

All claims expressed in this article are solely those of the authors and do not necessarily represent those of their affiliated organizations, or those of the publisher, the editors and the reviewers. Any product that may be evaluated in this article, or claim that may be made by its manufacturer, is not guaranteed or endorsed by the publisher.

## Supplementary material

The Supplementary material for this article can be found online at: https://www.frontiersin.org/articles/10.3389/fmicb.2022.986637/full#supplementary-material
