# Supplementary material for: Characterizing free-living and particle-attached bacterial communities of a canyon river reservoir on the Yungui Plateau, China
Source: Front Microbiol. 2022 Aug 31;13:986637. doi: 10.3389/fmicb.2022.986637 (PMC9470832; doi:10.3389/fmicb.2022.986637)
Supplement: Supplementary file 1 [file Data_Sheet_1.DOCX]

**Supplementary materials**


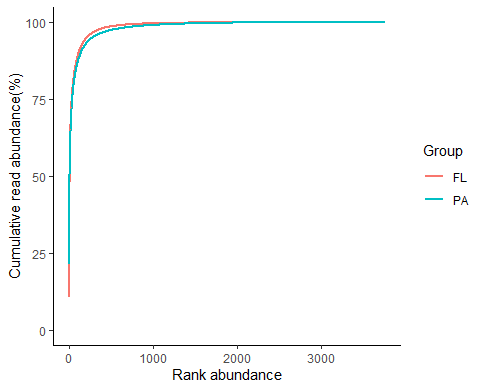


Fig. S1 Rank abundance curve of Free-living (FL) and Partcile-attached (PA) bacterioplankton in Wujiangdu Reservoir.


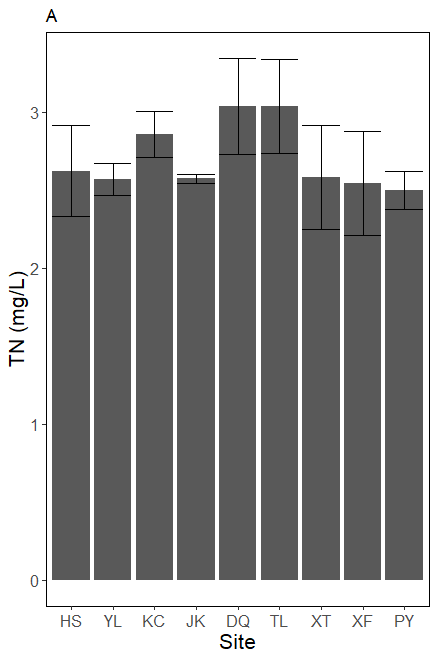

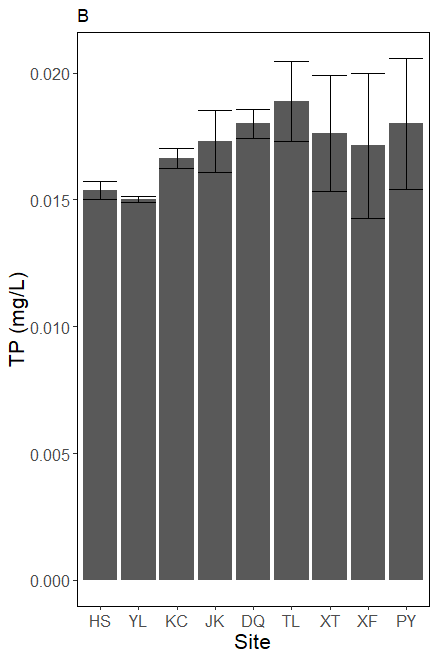

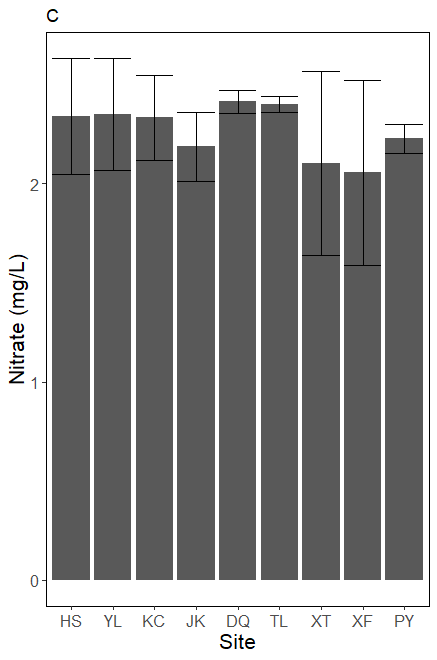

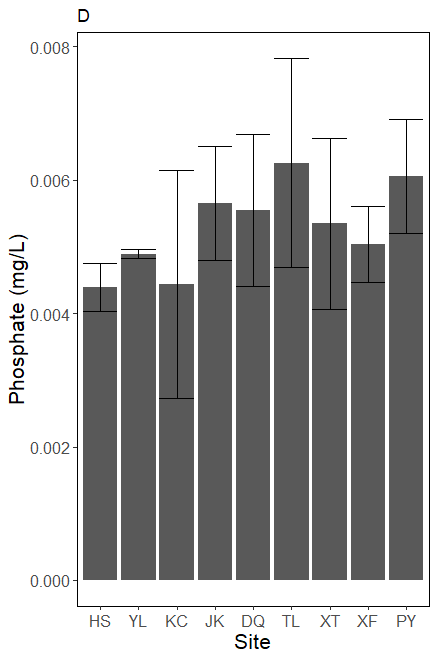

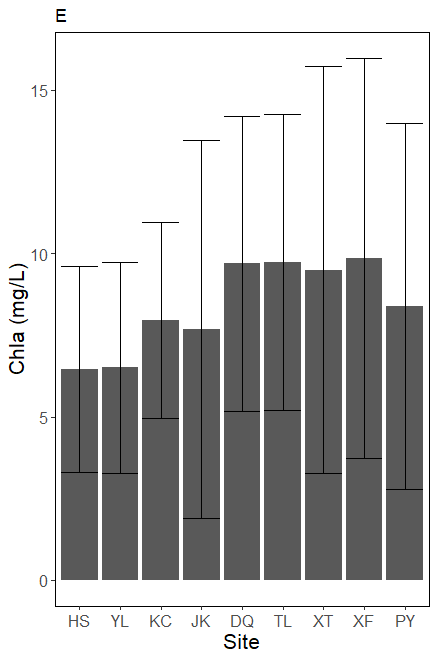

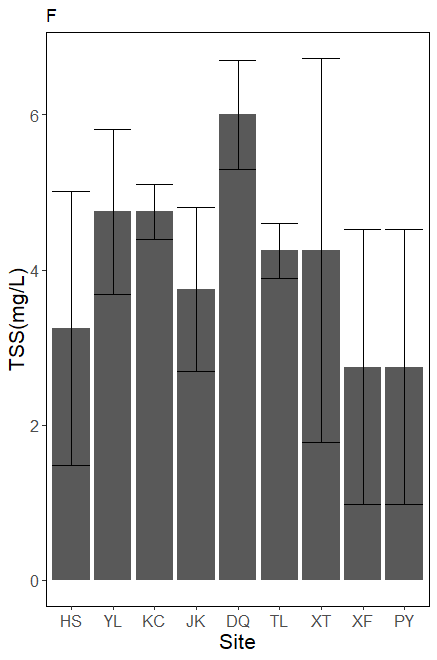


Fig. S2 Total nitrogen (A: TN), total phosphorus (B: TP), nitrate (C: NO_3_^-^), phosphate (D: PO_4_^3-^), Chlorophyll a (E: Chla), and Total suspended solids (F: TSS) concentrations in Wujiangdu reservoir.
